# Impact of the COVID-19 Pandemic Period on Patients with Head and Neck Carcinoma: A Systematic Review

**DOI:** 10.3390/diseases11020061

**Published:** 2023-04-10

**Authors:** Maria Carolina Pinto Pereira, Juliana Campos Hasse Fernandes, Gustavo Vicentis Oliveira Fernandes, Felipe Nor, Tiago Marques, Patrícia Couto

**Affiliations:** 1 Faculty of Dental Medicine, Universidade Católica Portuguesa, 3504-505 Viseu, Portugal; 2 Private Practice, Ann Arbor, MI 48109, USA; 3 Periodontics and Oral Medicine Department, University of Michigan School of Dentistry, Ann Arbor, MI 48109, USA; 4 Centre for Interdisciplinary Research in Health (CIIS), Faculty of Dental Medicine, Universidade Católica Portuguesa, 3504-505 Viseu, Portugal

**Keywords:** head and neck cancer, COVID-19, treatment, impact, life, consequences

## Abstract

Introduction: The COVID-19 pandemic has significantly impacted all public life and the global economy. Since its discovery, the disease has spread rapidly, which led to an unprecedented public health crisis and the adoption of extreme measures to limit community and hospital spread. As a result of a confluence of extraordinary circumstances caused by this pandemic, the doctrines of treatment for patients with head and neck carcinoma had to be reanalyzed, guaranteeing the well-being of both patients and health professionals as well as society itself. Objective: The aim of our systematic review was to study the impact of the COVID-19 pandemic period on head and neck cancer patients, the effects on the health care provided and on patient health. Materials and Methods: This systematic review was based on the PRISMA guidelines and PICO strategy, with the focus question, “How has the COVID-19 pandemic period conditioned the treatment of patients with head and neck carcinoma?” Thus, electronic research was carried out on six databases: LILACS, PubMed/MedLine, Web of Science, the Cochrane COVID-19 Study Register, Scielo, and Scopus, aiming to answer the research question by considering the objective and defined criteria. The following information was extracted: author and year of the publication, patients’ age, gender, time until the first appointment, time from the first appointment to the surgery, the period in the hospital, time in intensive care, TNM, general stage of cancer, diagnostic procedures, oncological procedures, reconstructive surgery, and postoperative complications. Results: Initially, 837 articles were found. After removing duplicates, we obtained 471 studies. After screening by title and abstract, 67 articles were selected for full-text reading (k = 92) in order to assess their eligibility. Thus, nine articles were included (k = 1.0). All data and statistical results were obtained and contrasted. The included studies made it possible to reveal distinct impacts felt in different institutions of several countries, not allowing generalizable conclusions to be drawn. However, some of the variables analyzed are worrying, namely, the limitations that occurred in some types of oncological surgeries, as well as the increase in the number of patients admitted with higher TNM classifications and more debilitated general conditions. Conclusion: Within the limitation of this review, the results showed efforts made to prevent the pandemic from affecting the healthcare provided. There were no significant differences in days inside the intensive care unit, postoperative complications, and, in most cases, length of stay in the hospital. There were no differences in the number of patients admitted with a history of recurrence or neoadjuvant treatment. However, some variables raise concerns, such as the increase in patients with more advanced stage and TNM classification and a decrease in certain oncological procedures.

## 1. Introduction

In December 2019, an outbreak of pneumonia of unknown origins broke out in the Chinese province of Wuhan, raising general concerns due to the ease of transmission. After numerous studies and after identification, this pathogen was named severe acute respiratory syndrome coronavirus 2 (SARS-CoV-2) by the Coronavirus Study Group, and the disease was named coronavirus disease 2019 (COVID-19) [1,2]. On 11 March 2020, the World Health Organization (WHO) declared COVID-19 as a pandemic, significantly impacting all public life, students, professionals, and the global economy [3,4].

Since its discovery, there has been a rapid spreading of the virus that has led to an unprecedented public health crisis and the adoption of extreme measures to limit community and hospital contamination [5]. Within a few weeks, the virus spread to other Asian countries, Europe, America, and finally to the whole world [6].

With a public health emergency continually testing the resilience of health systems around the world, health professionals must have quality evidence to identify risky behaviors, as well as prioritize resources where they are most needed [7]. This difficulty of balance between providing the patient with the necessary treatment and minimizing personal contact was felt in all medical practices. Many physicians from all areas and countries were faced with the difficult task of deciding which patients would be screened for surgery in the future and which patients would continue to be treated immediately [8].

Thus, head and neck surgeons were placed in a particularly difficult situation because head and neck cancers are heterogeneous pathologies that can not only be empirically managed: instead, they rely on a multidisciplinary approach. Furthermore, these are pathologies with rapid progression and, if treatments are not carried out, can increase the burden on health systems in the long term [8]. Thus, over the world, virtual consultations were prioritized, patient screening was implemented, as well as therapeutic adjustments and postponement of surgical treatments [9]. Hence, managing head and neck cancer can be difficult and challenging. In times of pandemic, the adversities are multiple and varied. It is important to understand the real impact of the COVID-19 pandemic on treatment paradigms and how the benefits of social isolation were balanced against the potentially deleterious effects of delays in interventions.

Thereby, the purpose of this systematic review was to understand and assess how the COVID-19 pandemic has conditioned the treatment of patients with head and neck cancer, mainly in terms of the effects felt on healthcare and on patients’ health. The relevance of this study is associated with the importance of understanding how the treatment needs were met without neglecting individual patients’ rights, safety, and well-being.

## 2. Materials and Methods

### 2.1. Study Design and Eligibility Criteria

This systematic review was based on the PRISMA guidelines and followed the PICO strategy to formulate the study question: “How has the COVID-19 pandemic period conditioned the treatment of patients with head and neck carcinoma?” The criteria applied are shown in Table 1:

### 2.2. Search Strategy

An electronic search was carried out in six databases: LILACS, PubMed/MedLine, Web of Science, the Cochrane COVID-19 Study Register, Scielo, and Scopus. Database searching was followed by a manual search on the Google search engine to identify the keywords associated with the topic of this systematic review. The following scientific terms were identified: “Oral Cancer”, “Head and Neck Cancer”, “Squamous Cell Carcinoma”, “COVID-19”, “Treatment”, “Management”, “Impact”, and “Effects”. Then, the following MeSH terms were applied: “Head and Neck Neoplasm”, “COVID-19”, “SARS-CoV-2”, and “Disease Management”. The MeSH terms were conjugated to free expression terms and Boolean operators as follows: (“COVID-19” [MeSH Descriptor Data 2021] OR “SARS CoV-2” [MeSH Descriptor Data 2021]) AND (“Head and Neck Neoplasms” [MeSH Descriptor Date 2021] OR “Head and Neck Cancer”) AND (“Disease Management” [MeSH Descriptor Data 2021] OR “Treatment” OR “Impact” OR “Effects”). Articles were filtered by period and by language (Portuguese, Spanish, and English).

### 2.3. Study Selection

The following data were extracted and inserted in tables: author and year of the publication, patients’ age, gender, time until the first appointment, time from the first appointment to the surgery, time period in the hospital, time in intensive care, TNM, general stage of cancer, diagnostic procedures, oncological procedures, reconstructive surgery, and postoperative complications.

## 3. Results

**Figure 1:**
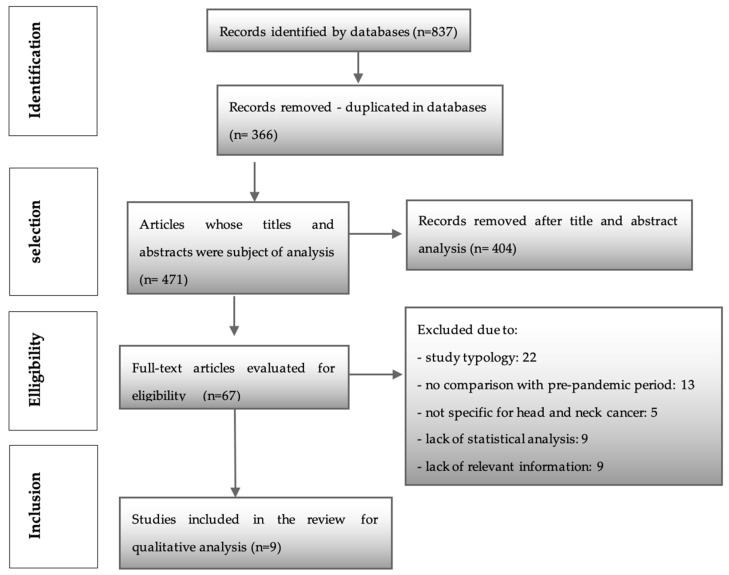
PRISMA flow chart.

After searching the above six databases, a total of 837 articles were obtained. Three hundred and sixty-six were identified as duplicates and were immediately removed. Then, 471 articles were assessed by two investigators (M.C.P.P. and P.C.). After analyzing titles and abstracts, this process resulted in the identification of 67 articles (k = 0.92), which followed through to a full reading and were assessed through the eligibility criteria. Of these, 22 articles were excluded due to the type of study. Thirteen did not make a comparison with the pre-pandemic period. Five were not specific to head and neck cancer, nine did not have relevant information, and nine articles were excluded due to statistical incompatibilities or lack of statistical analysis. Finally, nine studies were included for qualitative evaluation in this systematic review (Figure 1) (k = 1.0).

The main characteristics of the articles included are described in Table 2, including the author, year, and country, as well as the type of study, sample, objective, and statistical tests.

In order to compare the demographic characteristics of patients, data about the age and gender distribution of pre-pandemic and pandemic patients were analyzed, as well as the number of patients infected with COVID-19 (Table 3). To assess the possible pandemic impact on access to health care during the COVID-19 period, we evaluated the waiting time for hospital admission and surgery, as well as the days in intensive care and the length of stay in the hospital (Table 4 and Table 5).

Table 6 and Table 7 refer to the characterization of patients with head and neck carcinoma. The TNM classification, general disease stage, treatment history and recurrences were collected and evaluated. Naturally, these are clearly important variables to be analyzed, allowing the assessment of the tumor stage in which patients are presenting. The study of Tevetoglu et al. revealed a statistically significant increase in patients with T3–T4 tumors in the pandemic period, with more patients presenting considerably larger primary tumors above 2 cm. As for the involvement of regional lymph nodes, although there has been an increase in patients with regional metastases, it was not statistically significant [10]. Also, Lactourreye et al. [13] mention a statistically significant increase in patients with larger tumors (T3–T4) associated with greater lymph node involvement (N2–N3).

In studies by Kiong et al. and Batra et al., the results did not show statistically significant differences [12,17]. About the general stage of cancer, stages I and II define relatively small tumors without nodular involvement. Stages III and IV involve larger sizes of primary tumors, which can invade adjacent structures and/or spread to regional lymph nodes [19]. Thus, only Wai et al. revealed a statistically significant increase in patients with stages III and IV [11]. Neoadjuvant treatment presupposes a primary treatment prior to the main treatment.

None of the articles found significant differences in the number of patients admitted with a history of previous treatment [11,12,17]. Also, regarding patients with a history of recurrence, there were no statistically significant changes when comparing patients admitted in 2020 and 2019. When mentioning the impact of the pandemic period, it was especially important to analyze the effects on surgical procedures. Table 8 analyzes the total number of surgical procedures performed before and after the pandemic. In the same table, the diagnostic procedures are also evaluated. Reconstructive surgery data are analyzed in Table 9.

## 4. Discussion

All over the world, the need to adapt or limit medical practice was felt. These effects were felt with an impact on the specialty of the head and neck due to the peculiarities of this branch and the redirection of technical and human resources to combat COVID-19, which can delay the diagnosis and treatment of malignant pathologies. The pandemic situation is similar to other natural disasters, such as earthquakes or hurricanes. After Hurricane Katrina in 2005, there were notable delays in healthcare, and patients with head and neck cancer of all types of socioeconomic status found it difficult to obtain treatment as a result of the reduction in the capacity of hospitals by around 80% [20].

Thus, the immediate and long-term effects of the COVID-19 pandemic can last and remain for decades. Delays in care, diagnosis, investigation, and treatment may be involved, and the deleterious effect of delays in the treatment of patients with head and neck carcinomas, which are associated with worse survival rates, are already known, emphasizing the need to provide these individuals with adequate and timely treatment [21]. COVID-19 morbidity and mortality also seem to be associated with the burden of chronic diseases, the aging population, low responsiveness of health systems, and social inequalities, evidencing a syndemic effect [22].

All included studies tried to assess the consequences that the pandemic caused in patients with head and neck carcinoma. To allow for drawing stronger and more robust conclusions, analytical observational studies that made statistical comparisons with previous years were selected, which used the Chi-square U test, *t*-test, and/or Fisher’s test for categorical variables and the Mann–Whitney test for continuous variables, with values considered statistically significant when *p* < 0.05 [10,11,12,13,14,15,16,17]. In fact, there was an interesting number of analytical studies in the literature that assessed the impact of the pandemic period on the number of procedures performed, but a large part did not perform statistical analysis, making it less representative and less sensitive to small changes [18].

### 4.1. Impact of the Pandemic on Patients and Healthcare

The demographic characteristics of the patients, age and the proportion of male and female patients admitted in the pandemic and pre-pandemic periods were compared, and no statistically significant differences were found in any of the studies [10,11,12,13,17], with the exception of the studies published by Wai et al. and Riemann et al., whose sample of patients undergoing head and neck surgery during the pandemic was significantly older when compared to the previous year. The authors did not find any justification for this result regarding the age of the admitted patients [11,18].

Regarding the number of persons infected by COVID-19, this was a variable that naturally depended on the implemented testing capacity. Patients whose COVID-19 was detected in the preoperative test had their treatment postponed [10,13,17], always having considered that the delay must be adapted to the severity of the cancer [13]. Studies carried out by Tevetoglu et al., Akhtar et al., Salzano et al., and Batra et al. revealed that all patients undergo PCR testing before being admitted [10,15,16,17]. In some studies, tests were limited, as in the case of the study by Wai et al., in which only a survey about the symptoms was carried out and then some patients did a PCR test. Note that the study by Wai et al. refers to a pandemic period of only one month and in a very preliminary phase of the pandemic [11].

More focused on the impact of the pandemic on patients, some of the studies seek to assess the influence of the pandemic on the TNM classification and on the general stage of admitted patients. The article by Tevetoglu et al. demonstrated a statistically significant increase in patients with T3–T4 tumors when compared to the same period last year. However, they found no differences regarding regional lymph node metastases [10]. Lactourreye et al. found significant differences regarding both the size of the main tumor and the affectation of regional lymph nodes since T3–T4 and N2–N3 tumors were significantly more frequent in the pandemic period [13]. These findings allow us to unveil that there were already some countries, namely Turkey and France, in which the impact of the pandemic has been felt in patients present at the hospital since the number of patients with the disease in a more advanced phase was significant [10,13].

Regarding the general stage of the disease, there are two analytical studies that explored this issue: those by Wai et al. and Kiong et al. [11,12]. Only Wai et al. presented a significantly higher percentage of patients with more advanced stages of the disease (III and IV), even though no statistically significant differences were found regarding the TNM classification [11]. Another study showed that surgery for head and neck tumors should be scheduled in a timely manner due to the possible increase in tumors’ volume between one and three months, potentially compromising the airways and increasing the risk of bleeding, with delays resulting in disease progression [23]. These differences can be justified as an attempt by these countries to admit patients in an advanced locoregional state to avoid potential consequences on their individual survival [13].

Regarding surgeries, it is essential to assess the total surgical volume, which includes diagnostic surgeries, reconstructions, neck dissection surgeries, surgical ablations, transoral robotic surgery, and minor surgical procedures, such as surgical hemostasis or fistula closure. The studies of Wai et al., Laccourreye et al., Akhtar et al., and Salzano et al. compared the total number of surgeries with the pre-pandemic period [11,13,15,16]. The data were far from revealing homogeneity. In the study of Wai et al., there was a statistically significant increase in the number of transoral robotic surgeries and local reconstructions and a statistically significant decrease in the number of thyroidectomies, and all other procedures did not vary significantly [11]. In the study by Lactourreye et al., no significant variation was felt in the surgical volume, confirming the idea that these institutions in the Ile de France region managed to maintain some normality and stability in the treatment of patients without the pandemic having a significant impact [13]. The same did not happen in the studies by Akhtar et al. (India) and by Salzano et al. (Italy), which showed different conclusions. In both studies, there was a statistically significant increase in the proportion of head and neck surgical procedures performed in the 2020 period when compared to 2019 [15,16].

Another aspect that becomes important to compare is the reconstruction surgeries. Major oncologic surgeries of the head and neck often require reconstructive surgeries, more complex procedures, even in non-pandemic situations [24]. Four studies investigated the numbers of reconstructive surgeries [10,11,13,17], and three of those noted significant differences when comparing the numbers before and after COVID-19.

In the study by Tevetoglu et al., there was a significant increase in the number of reconstructive surgeries in cases of oral cancer and, in the study by Wai et al., in cases of head and neck cancer. In these studies, the increase in patients with more advanced cases was also statistically significant [10,11]. It is expected that the more advanced the stage or classification, the greater the surgical excisions and, therefore, the need for reconstruction [10].

On the other hand, Batra et al. revealed a different perspective, with a statistically significant decrease in the number of reconstructions performed [17]. Thus, it is worth noting that this was the only study in the pandemic period, although this difference was not statistically significant, that admitted a higher percentage of patients with T1–T2 tumors and therefore required fewer reconstructive surgeries since cases were selected earlier, to reduce both the time of surgery and to test the reliability and functioning of the designed system to face the pandemic [17].

Specifically, on diagnostic procedures, the study by Riemann et al. presented a statistically significant reduction in the number of diagnoses of suspected lesions, which the authors attributed to the discontinuation of follow-up/routine visits of the patients with head and neck cancer, which in addition to causing a decline in procedures and diagnoses, may result in a longer delay before the diagnosis of a recurrence [18]. The percentage of patients with recurrent disease and with a history of pre-surgical treatment admitted as well as postoperative complications during the pandemic period did not significantly differ when compared to the pre-pandemic period [11,12,13,15,17], revealing there was no change in the management of these patients.

About the variable “time between the first appointment up to the surgery”, only Wai et al. found significant changes, reporting a decrease in the waiting time from the first hospital admission to surgery, which is valid only in cases where the procedure performed was surgical ablation. It most likely resulted from the reflection of the decrease in patients treated for non-malignant pathologies, such as lipomas or sialadenitis [11]. About the “time until the first appointment”, Tevetoglu et al. found significant differences in the time elapsed from the first symptom to the first admission when comparing patients admitted in 2020 and in 2019. In the Turkish study, patients took longer to seek health care [10].

Regarding the length of stay in the hospital [11,13,14,17], there was no statistically significant difference in the length of stay, apart from the study by Batra et al., which attributed the increase to an average of three days of waiting time for a PCR test result, which in some circumstances was inconclusive and required repetition, increasing the length of stay in the hospital [17].

### 4.2. Limitations of the Study

One of the great difficulties felt in evaluating the impact of the pandemic on patients with head and neck cancer was being able to compare the data obtained in the articles due to a considerable heterogeneity, which is the result of such a recent topic. From the outset, the contemporaneity of the studies is also reflected in the reduced follow-up period, which may reflect seasonal variability and does not allow the analysis of crucial variables such as mortality and morbidity.

Also note the difficulty of generalizing results obtained in institutions, which may be manifestly different from the realities experienced in other situations even in the same country, since the results invariably depend on the number of COVID-19 cases in the region, the period analyzed and the resource availability. An important limitation of analytical studies is that most analyze patients admitted for surgery, not including information from patients who have had their treatments postponed. Thus, it is essential that subsequent studies assess longer follow-up periods, the largest possible number of institutions and that there is some agreement in the chosen time.

## 5. Conclusions

Within the limitation of this review, the results showed an effort so that the pandemic did not affect the healthcare provided. There were no significant differences in days inside the intensive care unit, postoperative complications, and, in most cases, length of stay in the hospital. There were no differences in the number of patients admitted with a history of recurrence or neoadjuvant treatment. However, some variables raise concerns, with an increase in patients with more advanced stage and TNM classification and a decrease in certain types of oncological procedures. These findings can be considered the beginning of the discovery of the “tip of the iceberg”, and the real consequences of this pandemic in cancer care may take a few months or even years to be discovered, with studies that assess the impact on mortality, morbidity, and quality of life of these patients. The true long-term effects of this pandemic will be evidenced as the normality returns and the impact on the diagnosis of new malignancies, recurrences, mortality, as well as the overall stage of patients seeking medical care is assessed.

## Figures and Tables

**Table 1:** Eligibility criteria applied.

Selection Criteria	Inclusion Criteria	Exclusion Criteria
Participants	Patients with head and neck carcinoma	Patients with other carcinomas
Interventions	Studies that analyze the impact of the pandemic on the treatment and management of these patients	Studies that analyze other variables
Comparisons	Comparison with the same period of previous years with *p*-values	Studies that do not compare with the pre-pandemic period
Outcomes	Influence of the pandemic on the way in which this pathology is treated and managed, presenting statistical analysis of the data	Studies that only assess the impact of the pandemic on the diagnosis of these patients; without statistical analysis
Study types	Controlled clinical trials, case-controls, experimental, quasi-experimental, analytical cross-studies, observational studies	Reviews, systematic reviews and meta-analysis, comments, expert opinion
Publication date	2020–2021	Before 2020
Languages	Portuguese, Spanish, English	Other languages

**Table 2:** General characteristics of the included articles.

Authors	Year	Country	Study Type	Sample	Objective	Statistical Tests
Tevetoglu et al. [10]	2021	Turkey	Retrospective observational study	56 patients were diagnosed and scheduled for head and neck surgery at the Tertiary Care Center from 15 March to 15 September 2020, compared to 60 patients scheduled in the same period of 2019.	Assess how COVID-19 affected patients with head and neck cancer.	Mann-Whitney U test Chi-square test
Wai et al. [11]	2020	USA	Retrospective observational study	83 patients were scheduled for cancer or reconstructive surgery at the Oncology Center of the University of California San Francisco from 16 March to 16 April 2020, compared to 56 patients scheduled in the same period in 2019.	Assess how the COVID-19 pandemic has affected health care for patients with head and neck cancer.	Chi-square test *t*-test
Kiong et al. [12]	2021	USA	Retrospective observational study	183 patients evaluated in MTM at the University of Texas from 14 March to 18 June 2020, compared to 252 patients evaluated in the homonymous period of 2019.	Assess the impact of the COVID-19 pandemic on healthcare and head and neck cancer patients.	Chi-square test Fisher’s exact test Mann-Whitney U test
Laccourreye et al. [13]	2020	France	Prospective observational study	118 patients were admitted to six ENT departments of university hospitals in the Ile de France region from 17 February to 17 March 2020, compared to 106 patients admitted from 18 March to 18 April.	Impact of the pandemic on the otolaryngology department of the university hospital in the Ile-de-France region, where the epidemic was most felt.	Chi-square test Fisher’s exact test Mann–Whitney U test
He et al. [14]	2021	China	Retrospective observational study	36 patients with head and neck cancer were treated at a radiotherapy center from 29 January to 11 April 2020, compared to 32 patients admitted to a radiotherapy center in the same period of 2019.	Compare information regarding patients admitted for radiotherapy before and after the pandemic.	Mann–Whitney U test Chi-square test Fisher’s exact test
Akhtar et al. [15]	2021	India	Retrospective observational study	248 patients were admitted for head and neck surgery in the oncology surgery department of an academic and university hospital in North India from April to September 2020, comparing 310 patients admitted in the same period in 2019.	Evaluate the performance of this cancer care center during the first six months of the pandemic.	Chi-square test
Salzano et al. [16]	2020	Italy	Retrospective observational study	113 patients were admitted for surgery at the ENT and maxillofacial cancer surgery department of the National Cancer Institute of Naples from 21 February to 25 May 2020, comparing 101 patients admitted in the same period in 2019.	Understanding the possible influence of the pandemic on the results of cancer surgery in patients with head and neck cancer.	*t*-test
Batra et al. [17]	2021	India	Prospective cohort study	21 patients underwent head and neck surgery at a tertiary care center in India between 19 May 2020, and 30 June 2020, compared to 193 patients who underwent surgery in the same period in 2019.	Identify trends and changes experienced in surgical practice compared to the pre-COVID period.	Pearson’s correlation coefficient
Riemann et al. [18]	2020	Germany	Observational study	Patients admitted for surgical procedures in the department of otolaryngology and head and neck surgery at Freiburg Hospital 8 weeks before and 8 weeks after 16 March 2020.	Estimating the collateral damage caused by the COVID-19 pandemic in ENT patients.	Mann–Whitney U test

**Table 3:** Demographic characteristics and number of patients infected with COVID-19.

Article	Average Age			Gender (M/F)			N of Patients with COVID-19
	Pre-Pandemic	Pandemic	*p*	Pre-Pandemic	Pandemic	*p*	n
Tevetoglu et al. [10]	61.2 ± 8.3 years	59.6 ± 7.7 years	0.47	nr	nr	nr	3
Wai et al. [11]	58 ± 15 years	63 ± 15 years	0.03	51/32	34/22	0.93	0
Kiong et al. [12]	64 years	65 years	0.747	121/35	87/30	0.538	nr
Laccourreye et al. [13]	64 years	63 years	0.92	80/38	79/27	0.3	3
Batra et al. [17]	47 years	49 years	0.58	165/28	14/7	0.06	1
Riemann et al. [18]	45.17 years	49.02 years	0.015	nr	nr	nr	nr

nr: not reported.

**Table 4:** Time from the first admission to the hospital and time from the first admission to surgery.

Article	Time until First Hospital Admission			Time from First Admission to Surgery		
	2019	2020	*p*	2019	2020	*p*
Tevetoglu et al. [10]	16.6 ± 5.9 weeks	19.01 ± 4.6 weeks	0.02	2.9 ± 1.2 weeks	3.4 ± 2.5 weeks	0.06
Wai et al. [11]	22 ± 50 days	9.7 ± 8.7 days	0.12	In cases of microvascular reconstruction ± ablation: 35 ± 23 days	14 ± 12 days	0.0002
Kiong et al. [12]	12 weeks	12 weeks	0.391	nr	nr	nr

nr: not reported.

**Table 5:** Length of stay at the hospital and days in Intensive Care Unit (ICU).

Article	Length of Stay (Days)			Number of ICU Days		
	Pre-Pandemic	Pandemic	*p*	Pre-Pandemic	Pandemic	*p*
Wai et al. [11]	In cases of microvascular reconstruction ± ablation: 7.3 ± 2.5	In cases of microvascular reconstruction ± ablation: 7.1 ± 2.5	0.87	0.63	0.86	0.33
	For ablation only: 2.2 ± 1.8	For ablation only: 2.9 ± 1.6	0.93			
Laccourreye et al. [13]	3	3	0.46	0	2	0.86
He et al. [14]	47.5	47	0.839	nr	nr	nr
Batra et al. [17]	7	10	0.001	nr	nr	nr

nr: not reported.

**Table 6:** TNM classification and general stage of cancer.

Article	TNM						Overall Stage		
	Pre-Pandemic		Pandemic				Pre-Pandemic	Pandemic	*p*
	T	N	T	*p*	N	*p*			
Tevetoglu et al. [10]	43 T1–T2	16N+	26 T1–T2	0.049	20 N+	0.29	n/a	n/a	n/a
	17 T3–T4		30 T3–T4						
Wai et al. [11]	19 T1–T2 (76%)	12 N+ (40%)	14 T1–T2 (55%)	0.14	12 N+ (44%)	0.91	I or II: 24 (80%)	I or II: 18 (53%)	(p = 0.02)
	2 T3–T4 (8%)		8 T3–T4 (30%)				III or IV: 6 (20%)	III or IV: 16 (47%)	
Kiong et al. [12]	Of 128 patients:	50N+ (46.7%)	Of 103 patients:	0.111	36N+ (46.2%)	0.656	I or II: 64 (60.9%) III or IV: 41 (39.0%)	I or II: 45 (60.8%) III or IV: 29 (39.2%)	0.782
	71 T1–T2 (66.6%)		42 T1–T2 (53.8%)						
	35 T3–T4 (32.7%)		31 T3–T4 (39.8%)						
Laccourreye et al. [13]	48 T1–T2	63 N0–N1 6N2–N3	28 T1–T2	0.002	40 N0–N1 21 N2–N3	0.0004	nr	nr	nr
	12 T3–T4		26 T3–T4						
Batra et al. [17]	8 T0 (4.1%)	95 N0 (49.2%) 98 N+ (50.8%)	12 T1–T2 (57.2%) 9 T3–T4 (42.8%)	0.09	9 N0 (42.9%) 12 N+ (57.1%)	0.28	nr	nr	nr
	57 T1–T2 (29.5%)								
	128 T3–T4 (66.4%)								

nr: not reported.

**Table 7:** Patients admitted with a history of pre-surgical treatment and a history of recurrences.

Article	Number of Patients with a History of Pre-Surgical Treatment			Number of Patients with a History of Recurrence		
	Pre-Pandemic	Pandemic	*p*	Pre-Pandemic	Pandemic	*p*
Wai et al. [11]	9 (11%)	3 (5%)	0.18	16 (34%)	12 (27%)	0.39
Kiong et al. [12]	28 (17.9%)	14 (12%)	0.175	31 (19.9%)	30 (25.6%)	0.257
Batra et al. [17]	71 (36.8%)	10 (47.6%)	0.35	nr	nr	nr

nr: not reported.

**Table 8:** Diagnostic procedures and total cancer procedures.

Article	Diagnostic Procedures			Total surgical procedures		
	Pre-Pandemic	Pandemic	*p*	Pre-Pandemic	Pandemic	*p*
Wai et al. [11]	6 (7%)	7 (11%)	0.4	Transoral Robotic Surgery: 6 (7%)	Transoral Robotic Surgery: 11 (17%)	0.05
				Thyroid: 19 (23%)	Thyroid: 1 (2%)	<0.0001
				Other Surgeries: 59	Other Surgeries: 51	>0.05
Laccourreye et al. [13]	47	44	0.81	71	62	0.54
Akhtar et al. [15]	nr	nr	nr	310 (52%)	248 (60%)	0.012
Salzano et al. [16]	nr	nr	nr	101	113	0.0011
Riemann et al. [18]	22/week	12/week	<0.005	nr	nr	nr

nr: not reported.

**Table 9:** Reconstructive surgeries and postoperative complications.

Article	Reconstructive Surgeries			Postoperative Complications		
	Pre-Pandemic	Pandemic	*p*	Pre-Pandemic	Pandemic	*p*
Tevetoglu et al. [10]	Total: 6	9	0.19	nr	nr	nr
	Only oral cavity: 5	8	0.024			
Wai et al. [11]	Microvascular Reconstruction ± ablation: 10 (12%)	Microvascular Reconstruction ± ablation: 15 (24%)	0.06	16 (19%)	14 (22%)	>0.05
	Local Reconstruction: 4 (5%)	Local Reconstruction: 9 (14%)	0.04			
Laccourreye et al. [13]	16	19	0.32	17	16	0.99
Akhtar et al. [15]	nr	nr	nr	27 (8%)	30 (12%)	0.114
Batra et al. [17]	115 (59.6%)	7 (33.3%)	0.03	nr	nr	nr

nr: not reported.

## References

[1] Harapan H., Itoh N., Yufika A., Winardi W., Keam S., Te H., Megawati D., Hayati Z., Wagner A.L., Mudatsir M. (2020). Coronavirus disease 2019 (COVID-19): A literature review. J. Infect. Public Health.

[2] Esakandari H., Nabi-Afjadi M., Fakkari-Afjadi J., Farahmandian N., Miresmaeili S.M., Bahreini E. (2020). A comprehensive review of COVID-19 characteristics. Biol. Proced. Online.

[3] Halboub E., AL-Maweri S.A., Al-Soneidar W.A. (2020). Utilization of COVID-19 testing for opportunistic screening of oral cancer. Oral. Oncol..

[4] dos Santos Gonçalves R.M., Fernandes G.V.O., Fernandes J.C.H., Seabra M., Figueiredo A. (2023). Impact of COVID-19 on Portuguese Dental Students: A Cohort Study. Healthcare.

[5] Han A.Y., Miller J.E., Long J.L., St John M.A. (2020). Time for a Paradigm Shift in Head and Neck Cancer Management During the COVID-19 Pandemic. Otolaryngology–Head Neck Surg..

[6] Kowalski L.P., Sanabria A., Ridge J.A., Ng W.T., de Bree R., Rinaldo A., Takes R.P., Mäkitie A.A., Carvalho A.L., Bradford C.R. (2020). COVID-19 pandemic: Effects and evidence-based recommendations for otolaryngology and head and neck surgery practice. Head Neck.

[7] Rod J.E., Oviedo-Trespalacios O., Cortes-Ramirez J. (2020). A brief-review of the risk factors for covid-19 severity. Rev. Saude Publica.

[8] Prasad A., Carey R.M., Rajasekaran K. (2020). Head and neck virtual medicine in a pandemic era: Lessons from COVID-19. Head Neck.

[9] Araujo S.E.A., Leal A., Centrone A.F.Y., Teich V.D., Malheir1 D.T., Cypriano A.S., Neto M.S., Klajner S. (2021). Impacto da COVID-19 sobre o atendimento de pacientes oncológicos: Experiência de um centro oncológico localizado em um epicentro Latino-Americano da pandemia. Einstein.

[10] Tevetoğlu F., Kara S., Aliyeva C., Yıldırım R., Yener H.M. (2021). Delayed presentation of head and neck cancer patients during COVID-19 pandemic. Eur. Arch. Oto-Rhino-Laryngol..

[11] Wai K.C., Xu M.J., Lee R.H., El-Sayed I.H., George J.R., Heaton C.M., Knott P.D., Park A.M., Ryan W.R., Seth R. (2021). Head and neck surgery during the coronavirus-19 pandemic: The University of California San Francisco experience. Head Neck.

[12] Kiong K.L., Diaz E.M., Gross N.D., Diaz E.M., Hanna E.Y. (2021). The impact of COVID-19 on head and neck cancer diagnosis and disease extent. Head Neck.

[13] Laccourreye O., Mirghani H., Evrard D., Bonnefont P., Brugel L., Tankere F. (2020). Impact of the first month of Covid-19 lockdown on oncologic surgical activity in the Ile de France region university hospital otorhinolaryngology departments. Eur. Ann. Otorhinolaryngol. Head Neck Dis..

[14] He J., Yang L., Tao Z., Yang J., Zhou Y., Wang R., Zhang Y., Huang Y., Zhou L., Sun B. (2021). Impact of the 2019 novel coronavirus disease (COVID-19) epidemic on radiotherapy-treated patients with cancer: A single-center descriptive study. Cancer Manag. Res..

[15] Akhtar N., Rajan S., Chakrabarti D., Kumar V., Gupta S., Misra S., Chaturvedi A., Azhar T., Parveen S., Qayoomet S. (2021). Continuing cancer surgery through the first six months of the COVID-19 pandemic at an academic university hospital in India: A lower-middle-income country experience. J. Surg. Oncol..

[16] Salzano G., Maglitto F., Guida A., Perri F., Maglione M.G., Buonopane S., Muto P., Ionna F. (2021). Surgical oncology of the head and neck district during COVID-19 pandemic. Eur. Arch. Oto-Rhino-Laryngol..

[17] Batra T.K., Tilak M.R., Pai E., Verma N., Gupta B.K., Yadav G., Dubey R.K., Francis N.J., Pandey M. (2021). Increased tracheostomy rates in head and neck cancer surgery during the COVID-19 pandemic. Int. J. Oral. Maxillofac. Surg..

[18] Riemann S., Speck I., Gerstacker K., Becker C., Knopf A. (2020). Collateral damage of the COVID-19 pandemic: An alarming decline in critical procedures in otorhinolaryngology in a German university hospital. Eur. Arch. Oto-Rhino-Laryngol..

[19] National Comprehensive Cancer Network (2020). Head and Neck Cancers.

[20] US Government Accountability Office (2006). Status of the Healthcare System in New Orleans.

[21] Yan F., Knochelmann H.M., Morgan P.F., Kaczmar J.M., Neskey D.M., Graboyes E.M., Nguyen S.A., Ogretmen B., Sharma A.K., Day T.A. (2020). The evolution of care of cancers of the head and neck region: State of the science in 2020. Cancers.

[22] Barbosa T.P., da Costa F.B.P., Ramos A.C.V., Berra T.Z., Arroyo L.H., Alves Y.M., Santos F.L.D., Arcêncio F.L.A. (2022). COVID-19 morbidity and mortality associated with chronic disorders, healthcare services, and inequity: Evidence for a syndemic. Rev. Panam. Salud Publica.

[23] Jensen A.R., Nellemann H.M., Overgaard J. (2007). Tumor progression in waiting time for radiotherapy in head and neck cancer. Radiother. Oncol..

[24] Kiong K.L., Guo T., Yao C.M.K.L., Gross N.D., Hanasono M.M., Ferrarotto R., Rosenthal D.I., Myers J.N., Hanna E.Y., Lai S.Y. (2020). Changing practice patterns in head and neck oncologic surgery in the early COVID-19 era. Head Neck.

